# Hippo Signaling: Emerging Pathway in Stress-Related Psychiatric Disorders?

**DOI:** 10.3389/fpsyt.2018.00715

**Published:** 2018-12-21

**Authors:** Jens Stepan, Elmira Anderzhanova, Nils C. Gassen

**Affiliations:** ^1^Department Translational Research in Psychiatry, Max Planck Institute of Psychiatry, Munich, Germany; ^2^Clinic and Polyclinic of Psychiatry and Psychotherapy, Bonn University Clinic, Bonn, Germany

**Keywords:** hippo pathway, KIBRA, psychophysiological stress, synaptic plasticity, glucocorticoids, GPCRs

## Abstract

Discovery of the Hippo pathway and its core components has made a significant impact on our progress in the understanding of organ development, tissue homeostasis, and regeneration. Upon diverse extracellular and intracellular stimuli, Hippo signaling regulates stemness, cell proliferation and apoptosis by a well-conserved signaling cascade, and disruption of these systems has been implicated in cancer as well as metabolic and neurodegenerative diseases. The central role of Hippo signaling in cell biology also results in prominent links to stress-regulated pathways. Genetic variations, epigenetically provoked upregulation of Hippo pathway members and dysregulation of cellular processes implicated in learning and memory, are linked to an increased risk of stress-related psychiatric disorders (SRPDs). In this review, we summarize recent findings, supporting the role of Hippo signaling in SRPDs by canonical and non-canonical Hippo pathway interactions.

## Introduction

When the Hippo pathway was first discovered in Drosophila, it appeared as a linear kinase cascade highly relevant for proliferation and homeostasis, because deletion of core component genes resulted in an uncontrolled growth of multiple tissues (1, 2). Subsequent research identified mammalian orthologs of Hippo components and additional kinases, transcription factors and various adapter proteins directly or indirectly involved in Hippo signaling, providing a complex molecular network with strong regulatory effects on development, homeostasis, and regeneration (3–5). Upstream activators of the Hippo pathway include G-protein-coupled receptors (GPCR), integrins, and cell-cell adhesion factors, stress-reactive glucocorticoid hormones, metabolism-regulating hormones, growth factors, and mitogens (6).

Dysregulated Hippo signaling is associated with various cancers and a wide range of metabolic, cardiovascular, neurodevelopmental, and neurodegenerative diseases (3, 7). Regulators of Hippo pathway are expressed in the adults' brain suggesting their implementation in normal brain performance. Recent research further extends the Hippo signaling network and its potential to be therapeutically harnessed based on genetic association studies linking Hippo pathway members to stress-related-psychiatric disorders (SRPDs) (8–11). Key molecular and cellular processes that are thought to be involved in the pathophysiology of SPRDs are modulated by Hippo pathway members. Furthermore, various proteins of the Hippo signaling pathway are linked via the GR, GPCRs, Wnt-signaling and other pathways to stress-regulated signaling cascades (12–16).

In this review we highlight emerging evidence of an interaction between Hippo signaling and the stress axis and suggest how this novel link may correlate with the genesis of SRPDs.

## The Hippo Pathway in Mammals and Its Canonical Activation

**Figure 1:**
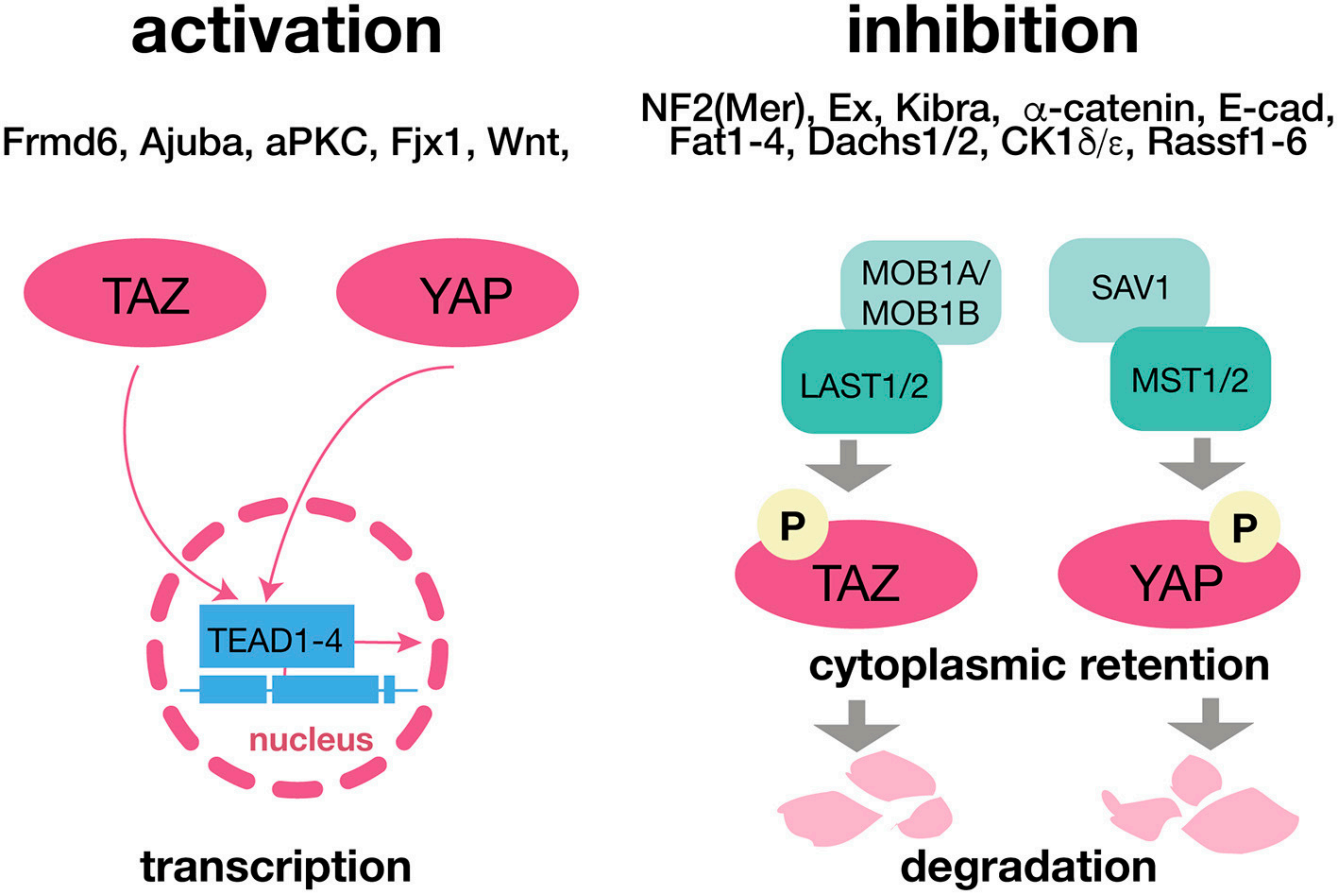
The Hippo Pathway and its canonical upstream regulators. The translocation of YAP and TAZ and respective translational effects of the Hippo pathway are omitted when these two factors are phosphorylated due to LAST1/2 and MST1/2 activity. Both components can be affected independently via a wide range of canonical upsream regulators. Being retained in the cytoplasm YAP and TAZ are ubiquitinated and degraded. ADHD, attention deficit hyperactivity disorder. LATS 1/2, large tumor suppressor kinase 1/2; MOB1A/MOB1B, Mps one binder kinase activator 1A/1B; MST1/2, macrophage-stimulating protein 1/2; SAV1, salvador family WW domain containing protein 1 (protein WW45); TAZ, transcriptional co-activator with PDZ-binding motif; TEAD1-4, TEA domain transcription factors 1-4; YAP, yes-associated protein.

The regulatory endpoints of the Hippo pathway are the two homologous transcriptional co-activators, yes-associated protein (YAP) and transcriptional co-activator with PDZ-binding motif (TAZ) (3, 7) (Figure 1). YAP and TAZ are widely expressed throughout the brain and non-neuronal tissues especially during embryogenesis^1^ In adult humans YAP is expressed in the subventricular zone of the lateral ventricle and subgranular zone of the dentate gyrus, the regions providing neurogenesis in mammalian brains. Weak immunostaining was found in the prefrontal cortex of humans (17). YAP expresses in the midbrain, possibly, to protect dopaminergic neurons from degeneration (18). TAZ expression appears to contribute to brain mitochondrial respiration, the function of hippocampal neurons and glia, and modulates cognitive abilities in mice (19). It is of note that Hippo pathway activity is retained in the adult hippocampus. A role of the hippocampus in neurogenesis and stress resilience (20), denote the Hippo pathway as a target for biomarker discovery and therapeutic interventions in SRPDs.

The regulation of YAP and TAZ is governed by two major protein kinase complexes, the mammalian Sterile 20-like kinases 1 and 2 (MST1/2), and the large tumor suppressor homolog LATS1/2 and their direct interaction partners SAV1 (MST1/2) and MOB1A/MOB1B (LATS1/2). Activation of MST1/2 and LATS1/2 causes phosphorylation of YAP/TAZ. Phospho-YAP/TAZ is either degraded or sequestered in the cytoplasm by the 14-3-3 protein, whereas after inactivation of the upstream kinase cascade dephosphorylated YAP/TAZ translocate to the nucleus. AJUBA antagonizes YAP phosphorylation and therefore prevents its activation. Through association with various transcription factors, like the TEAD family transcription factors (TEAD1-4), YAP/TAZ initiates transcription of several genes mainly involved in the regulation of development, homeostasis, and regeneration (3, 7) (Figure 1). This core-signaling cascade is activated/ inactivated by multiple stimuli and modulated by various post-translational modifications or through hetero complex re-organization, e.g., NF2 (Merlin) inhibits LATS through phosphorylation (3, 7) (Figure 1). Although, YAP and TAZ are primarily controlled at the level of their nuclear accumulation (nucleocytoplasmic shuttling), it is incompletely elucidated if nuclear entry occurs passively (diffusion), if it is a mediated process, or a combination of both (21). In a recent report mechanical forces have been shown to increase the permeability of the nuclear pore thereby facilitating the nuclear accumulation of YAP (22), whereas another study identified a nuclear localization sequence (NLS) and a nuclear export sequence (NES) for TAZ (21). Moreover 14-3-3 protein and TEAD family members have been proposed to be cytosolic and nuclear “retention factors,” respectively (21).

## Non-canonical Regulation of Hippo Signaling by Psychophysiological Stress

Accumlating evidence suggests that the core complexes and accessory proteins of the Hippo pathway can be modulated by molecular pathways that play a fundamental role in stress signaling. The non-canonical regulation of the Hippo pathway with regard to SRPDs will be the focus of the following chapter.

## Glucocorticoids Impact on Hippo Pathway

Release of glucocorticoids (GCs), such as cortisol, from the adrenal glands, is the final stage of hypothalamic-pituitary-adrenal (HPA) axis activation during emotionally stressful experiences [psychophysiological stress, depicted as “stress” throughout the manuscript, (23)]. GCs belong to the class of steroid hormones and act via specialized nuclear receptors to adapt behavior to a constantly changing environment. Despite the critical role that stress plays for body homeostasis, it is widely implicated in the onset of SRPDs (23). Sorrentino and colleagues described a molecular cascade that links glucocorticoid signaling to YAP regulation. In an interdisciplinary approach the researchers show, that the activation of glucocorticoid receptors (GRs) results in elevated YAP protein levels, its translocation to the nucleus and subsequently to enhanced transcriptional activity. Fibronectin was identified as a target of the GR. Increased fibronectin expression stimulates the focal adhesion-Src pathway, which in turn activates cytoskeleton-dependent YAP activation providing a direct link between the stress-hormone axis and Hippo signaling (24).

## GPCRs and Hippo Pathway in SRPDs and Related Psychopathologies

### GPCR Signaling

Extracellular signals act on synapses to drive spine morphogenesis and synaptic plasticity. Among multiple classes of receptors G protein-coupled receptors (GPCRs) are the working horses of neuronal communication. Overexpression or exogenous stimulation of a variety of GPCRs corresponds to Hippo pathway activity. Serotonin 5-HT4, adrenerergic α1B, metabotropic glutamate mGlu2, and adenosine A1A receptors are directly mediating neuronal transmission in the brain and are shown to contribute to stress-related abnormalities in mammals (25–29). These receptors, which are linked to brain-body crosstalk (LPA receptors, purinergic receptors, muscarinic acetylcholine receptor M1, angiontensin II receptor, free fatty acid receptor 1, platelet-activating factor receptor, thromboxane A2, frizzled homolog D4, complement component 3a receptor 1, estrogen receptor 1, opioid receptor Δ1, secretin receptor, thyroid-stimulating hormone receptor, gastrin-releasing peptide receptor, melanocortin receptor 1, somatostatin receptor 1, prostaglandin E receptor 2, and bombesin-like receptor 3) affect both the Hippo-YAP and Hippo-TAZ signaling via activation of Rho GTPases (16).

In contrast, dopamine D1 and adrenergic β2 receptors appear as a way for the selective inhibition of Hippo-YAP signaling. These GPCRs induce YAP phosphorylation mainly via cAMP and PKA (16).

Selective regulation of the Hippo-YAP signaling by 5-HT2B receptors activation has been shown in hepatocytes (30) and cardiomyocytes (31). These data suggest an effect of acute and chronic serotonin neurotransmission disturbance on Hippo signaling and provides a strong link between stress and related pathologies in peripheral organs. Although most available drugs to treat the symptoms of SRPDs (antidepressants) target serotonergic neurotransmission (32), a putative modulation of Hippo signaling by antidepressants remains a topic of future research.

## Wnt Signaling

Components of the Wnt pathway are transcriptional targets and therefore downstream targets for the Hippo pathway (13–15). The upstream influence of the canonical Wnt/β-catenin signaling on the Hippo pathway has been described recently (12). Consequently, a dynamical interaction in the presence of Wnt YAP/TAZ is released from the destruction complex, escaping degradation in the cytoplasm. In absence of Wnt the YAP/TAZ-dependent β-TrCP (β-transducin repeats-containing proteins) recruitment allows β-catenin destruction (33). Notably, the β-TrCP-mediated β-catenin degradation is GSK3–dependent (34). GSK3 plays a critical role in the regulation of Wnt—Hippo interaction (14).

## Canonical Hippo Pathway Links to SRPDs

Multiple studies have shown a direct association between members of the hippo pathway and SRPDs. Most data comes from genetic studies that report an association of allelic variation in the KIBRA (KIdney and BRAin) gene with (episodic) memory performance, gray and white matter volume and differences in functional brain activity (35–41). Substitution of C for T in the 9th intron (rs17070145) of the KIBRA gene, was first linked to memory performance and functional brain activity in a genome-wide association study (35). However, the functional role of the gene is still unclear since replication of the first results has proven difficult and sometimes delivered contradicting results. In line with the initial results, the rs17070145-T allele has been associated with better episodic memory functioning (36–41). However, several other studies have either associated the absence of rs17070145-T with better memory performance (42, 43), or were unable to show any link of this Single Nucleotide Polymorphism (SNP) with cognitive capabilities (43–46). CLSTN2 (calsyntenin 2), another hippo pathway member (SNP rs6439886), is mainly localized in the postsynaptic compartment of excitatory neurons in brain regions relevant for learning and memory like the medial temporal lobe (47), and has also been linked to memory performance by Passotiropoulos et al. (35) and in subsequent cohorts (48, 49). Another study, however, did not support the influence of the KIBRA SNP, with or without the CLSTN2 SNP, on longitudinal memory decline or hippocampal atrophy in older adults (44).

It has been speculated, that the lack of consensus across studies stem from age-related neuropathological changes on memory performance, which may interact with polymorphisms such as KIBRA and CLSTN2, the so-called “resource modulation hypothesis” (40, 44). Supporting evidence comes from studies taking age, increased risk for specific diseases and pre-existing diseases into account (9, 10, 46). Stickel et al. (40) report, that KIBRA results in decreased verbal memory performance and lower brain volumes in CC homozygotes compared to T carriers, particularly among older persons (40). In individuals with unipolar depression, Pantzar et al. (10) showed an interactive effect of KIBRA and CLSTN2 polymorphisms on memory performance, but not in older individuals without depression (10). They also found poorer episodic recall and recognition performance in non-T carriers (10). In contrast, in patients with major depressive disorder, Liu et al. (9) found that rs17070145 associates with better memory performance in non-T carriers (9). In cognitively normal adults with different genetic risk of Alzheimer's disease, based on their Aβ-amyloid levels and apolipoprotein E (APOE) ε2/ε3/ε4 genotype, Porter et al. (46) reported faster rates of cognitive decline and hippocampal atrophy in individuals with higher Aβ-amyloid levels and APOE ε4 + ve, that did not carry the rs17070145-T allele (46). Although this suggests that the exact role of the KIBRA, SNP rs17070145 in learning and memory is still unclear, further investment in understanding its well-established role in cognitive performance is essential to make progress from mechanism to disease in SRPDs.

Another association of two neighboring SNPs in the KIBRA gene in almost complete linkage disequilibrium, rs10038727, and rs4576167, with lifetime risk for post-traumatic stress disorder was described in two samples from African conflict regions (8). Carriers of the minor allele of both SNPs displayed a diminished disease risk (8). Nitric oxide synthase 1 adaptor protein (NOS1AP) also known as carboxyl-terminal PDZ ligand of neuronal nitric oxide synthase protein (CAPON) is an adaptor protein of the Hippo pathway and is encoded by the NOS1AP gene in humans (11, 50). CAPON is supposed to modulate glutamate neurotransmission via interaction with postsynaptic density (PSD) scaffolding proteins PSD93 and PSD95 (50). Xu et al. (11) showed an increased expression of CAPON in the prefrontal cortex in post-mortem tissue of patients with bipolar disorder (11).

## KIBRA as Potential Mediator of Synaptic Stress Effects

Accumulating evidence suggests that the scaffold protein expressed by the KIdney and BRAin gene [KIBRA; sometimes referred to as WW and C2 domain-containing protein 1 (WWC1)], is critical for synaptic plasticity, the cellular mechanism thought to underlie learning and memory (51–56). Althought it has not yet been demonstrated directly, KIBRA is a potential candidate to, at least partially, mediate the well-established stress effects on synaptic plasticity and cognitive performance (57, 58).

KIBRA is predominantly expressed in the kidney and the brain, in particular in structures important for learning and memory like the hippocampus, cortex, cerebellum, and hypothalamus (59, 60). In neuronal cells, KIBRA has a somatodendritic staining pattern with enrichment in perinuclear regions and the postsynaptic density (PSD) (54, 59). Previous studies have shown that KIBRA has various bindings partners, mainly mediated by the two N-terminal WW domains, a glutamic acid–rich motif and motifs for binding atypical PKC and PDZ domains (54, 56, 61). This includes the postsynaptic proteins dendrin and synaptopodin, postsynaptic α-amino-3-hydroxy-5-methyl-4-isoxazolepropionic acid receptors (AMPARs, the main fast stimulatory receptor of the neurotransmitter glutamate), and the atypical protein kinase C (PKC) isoform protein kinase Mζ (PKMζ) (52, 53, 59, 60, 62, 63).

PKMζ is brain specific and crucially involved in AMPA-receptor trafficking, a core mechanism of synaptic plasticity, and in the maintenance of long-term potentiation (LTP) in the hippocampus, which is thought to be the cellular correlate of learning and memory in mammals and involves AMPA and NMDA receptors of glutamate (51, 63–65). PKMζ is colocalized with KIBRA especially in the hippocampus and dentate gyrus (65), and KIBRA knock-out mice exhibit reduced learning and memory performance in spatial memory tasks, accompanied by decreased PKMζ levels (56). These results are in line with the observation that KIBRA associates with AMPARs and its partner protein interacting with C-kinase 1 (PICK1), which has been shown to accelerate the rate of AMPAR subunit recycling to the postsynaptic membrane (53). Moreover, KIBRA knock out mice exhibit an impaired LTP and long-term depression (LTD) in the hippocampus and show deficits in contextual fear learning and memory (53).

Overexpression of KIBRA in neurons facilitates LTP, but prevents the induction of LTD, likely by an increased constitutive recycling of AMPARs. In contrast, knock down of KIBRA abolishes LTP and decreases AMPAR recycling supporting a role of KIBRA as a bidirectional regulator of synaptic plasticity in hippocampal neurons (52). In a recent study, Tracy et al. (55) show that memory loss and LTP impairment in a mouse model of Alzheimer's disease critically depends on reduced synaptic KIBRA levels accompanied by reduced activity-induced postsynaptic actin remodeling and AMPAR insertion, which can be rescued by promoting actin polymerization or by restoring KIBRA expression (55).

The WWC family comprises two additional highly similar paralogs, WWC2, and WWC3 (61). Although it has been speculated that WWC2 can balance WWC1 knock out (53), their role in brain function remains unclear.

## Conclusion

**Figure 2:**
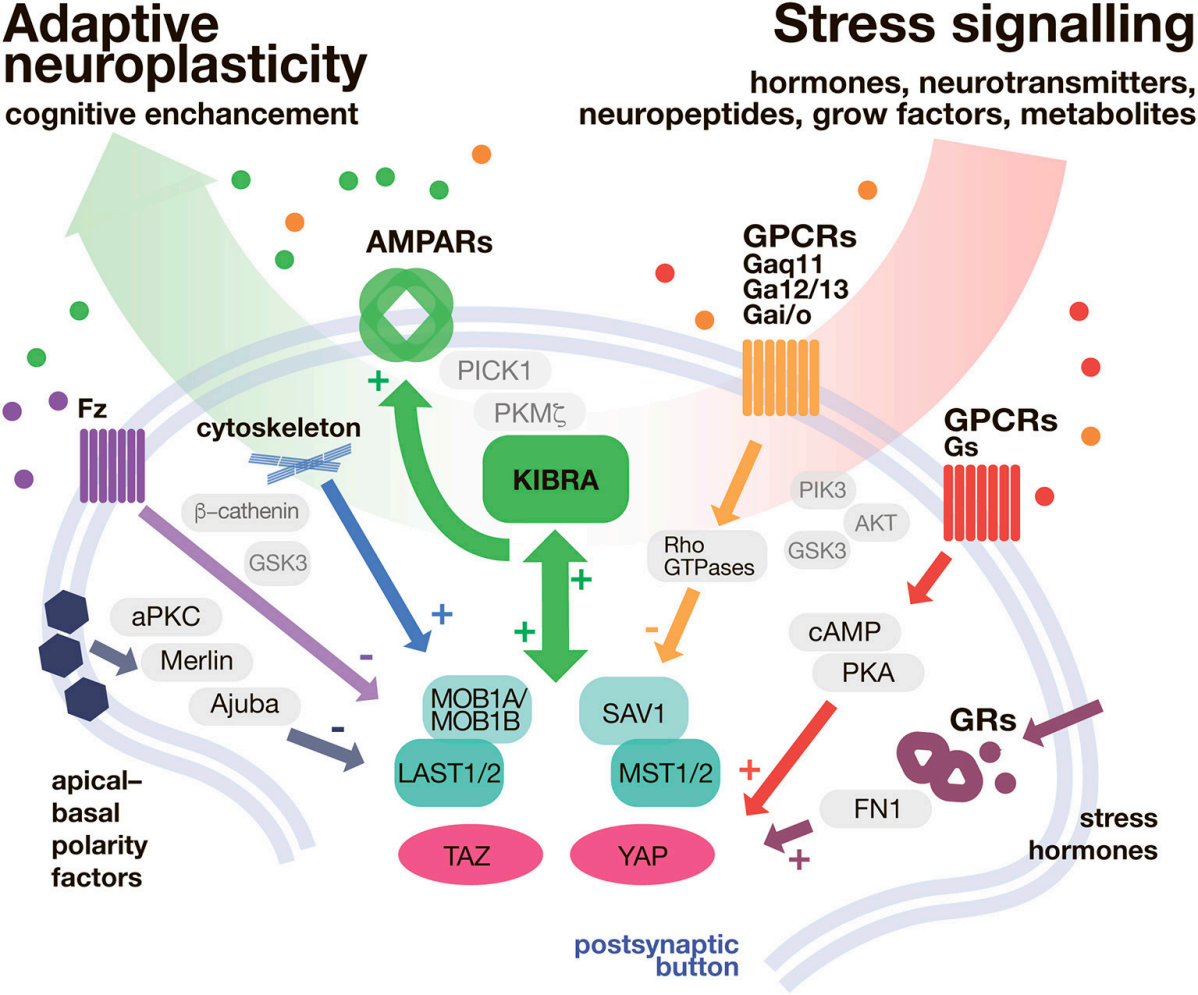
KIBRA/Hippo pathway as a shunt of stressful input. In the postsynaptic button Hippo pathway is modulated with a variety of secondary messenger systems, implemented in transduction of neurontransmitters, neuropeptides, and hormones. The Hippo pathway biderectioinally interacts with KIBRA signaling. In turn, the adaptive, neuroplasticity determined by AMPAR expression (and trafficking) is promoted. AMPARs, α-amino-3-hydroxy-5-methyl-4-isoxazolepropionic acid receptors; cAMP, cyclic adenosine monophosphate; FN1, fibronectin 1; Fz, Frizzeled; GSK3, Glycogen synthase kinase 3; GPCR, G protein coupled receptors; GR, glucocorticoid receptor; KIBRA, Kidney and Brain Protein 1 (also WWC1); LATS 1/2, large tumor suppressor kinase 1/2; MOB1A/MOB1B, Mps one binder kinase activator 1A/1B; MST1/2, macrophage-stimulating protein 1/2; PICK1, protein interacting with C-kinase 1; PKA, protein kinase A; PKC, protein kinase C; Rho GTPase, Rho guanosine-5′-triphosphatase; SAV1, salvador family WW domain containing protein 1 (protein WW45); TAZ, transcriptional co-activator with PDZ-binding motif; YAP, yes-associated protein.

Strong evidence suggests that both, Hippo- and stress signaling are involved in the pathophysiology of SRPDs. However, the possible interaction between Hippo signaling and the stress hormone axis has been widely neglected so far. Especially KIBRA as a mediator of adaptive neuroplasticity that is directly linked to the stress hormone axis via GR-signaling might balance the reduced cognitive capabilities observed in most SRPDs (see Figure 2).

Although there are many important questions that remain unanswered (e.g., exact role of KIBRA in memory), pharmacological targeting of Hippo signaling might offer guidance for the development of novel prophylactic and therapeutic approaches to treat SRPDs more effectively.

## Author Contributions

All authors listed have made a substantial, direct and intellectual contribution to the work, and approved it for publication.

### Conflict of Interest Statement

The authors declare that the research was conducted in the absence of any commercial or financial relationships that could be construed as a potential conflict of interest.

## Footnotes

^1^Mouse Brain Atlas. http://mousebrain.org/genesearch.html
